# Recurrent fainting while seated solved: a rare case of olanzapine-induced non-orthostatic syncope in a young male

**DOI:** 10.1093/ehjcr/ytaf462

**Published:** 2025-09-30

**Authors:** Jorge L Reyes, Ciana Keller, Thaddeus Walczak, Emanuel Ebin, David G Benditt

**Affiliations:** Cardiovascular Division, Department of Medicine, University of Minnesota Medical School, 420 Delaware Street SE, Minneapolis, MN 55455, USA; Department of Medicine, University of Minnesota Medical School, Minneapolis, MN, USA; Department of Neurology, University of Minnesota Medical School, Minneapolis, MN, USA; Cardiac Electrophysiology, Mercy Medical Center, 4050 Coon Rapids Blvd, Coon Rapids, MN 55433, USA; Cardiovascular Division, Department of Medicine, University of Minnesota Medical School, 420 Delaware Street SE, Minneapolis, MN 55455, USA

**Keywords:** Case report, Syncope, Orthostatic hypotension, Olanzapine

## Abstract

**Background:**

Olanzapine is an antipsychotic agent with alpha-1-adrenergic receptor blockade properties. While olanzapine therapy has been associated with orthostatic hypotension. symptomatic hypotension unrelated to increased postural stress has not been previously reported.

**Case summary:**

A 24-year-old male, newly initiated on the antipsychotic olanzapine, presented with new-onset multiple apparent near-syncope and syncope episodes often occurring while he was seated. Physical and neurologic exams were normal. complete bood count, basic metabolic panel, and urine drug testing were unremarkable. Baseline electroencephalogram (EEG) and head CT were normal. Echocardiogram revealed preserved biventricular function with left ventricular ejection fraction 60%–65%. Comprehensive autonomic testing, including multi-channel ECG recording, beat-to-beat blood pressure monitoring, and simultaneous EEG, documented multiple symptomatic paroxysmal hypotensive episodes occurring while the patient remained seated, without associated bradyarrhythmia or tachyarrhythmia. EEG slowing (delta waves) consistent with cerebral hypoperfusion accompanied the hypotensive episodes. In the absence of an alternative explanation, olanzapine was discontinued, and the symptoms resolved over the following weeks, supporting an olanzapine-induced aetiology.

**Discussion:**

The antipsychotic olanzapine may trigger hypotension without orthostatic stress or evident arrhythmia, suggesting an accentuated vasodepressor effect consistent with olanzapine’s known alpha-adrenergic blocker properties. Thus, olanzapine is a potential cause of intermittent, non-orthostatic hypotension; dose reduction or discontinuation should be considered in such cases.

Learning points

- In patients with recurrent syncope, a thorough clinical evaluation should always include a review of recently initiated medications.
- Olanzapine, an antipsychotic with alpha-1-adrenergic receptor blockade properties, can rarely cause non-orthostatic hypotension, alongside its known orthostatic effects.
- In instances of unexplained syncope in individuals being treated with olanzapine, consider dose reduction or olanzapine discontinuation.

## Introduction

Olanzapine is an atypical antipsychotic frequently prescribed for a range of psychiatric disorders, including schizophrenia and bipolar disorder. Its therapeutic effects are primarily attributed to its modulation of dopamine and serotonin receptors, but it also has alpha-1-adrenergic antagonist properties (*Summary figure*).^1,2^ The latter effect can lead to a reduction in vascular tone and may result in orthostatic hypotension (OH), commonly manifesting as dizziness upon transitioning from a supine to a seated or standing position. While OH is a recognized side effect of olanzapine, we present a previously undocumented case of non-orthostatic hypotension leading to syncope while seated, an adverse effect not previously documented in the literature.

## Summary figure

Mechanism and clinical timeline of olanzapine-induced non-orthostatic syncope, highlighting alpha-1 adrenergic antagonism-mediated vasodilation leading to decreased cerebral perfusion and syncope while seated.

**Figure:**
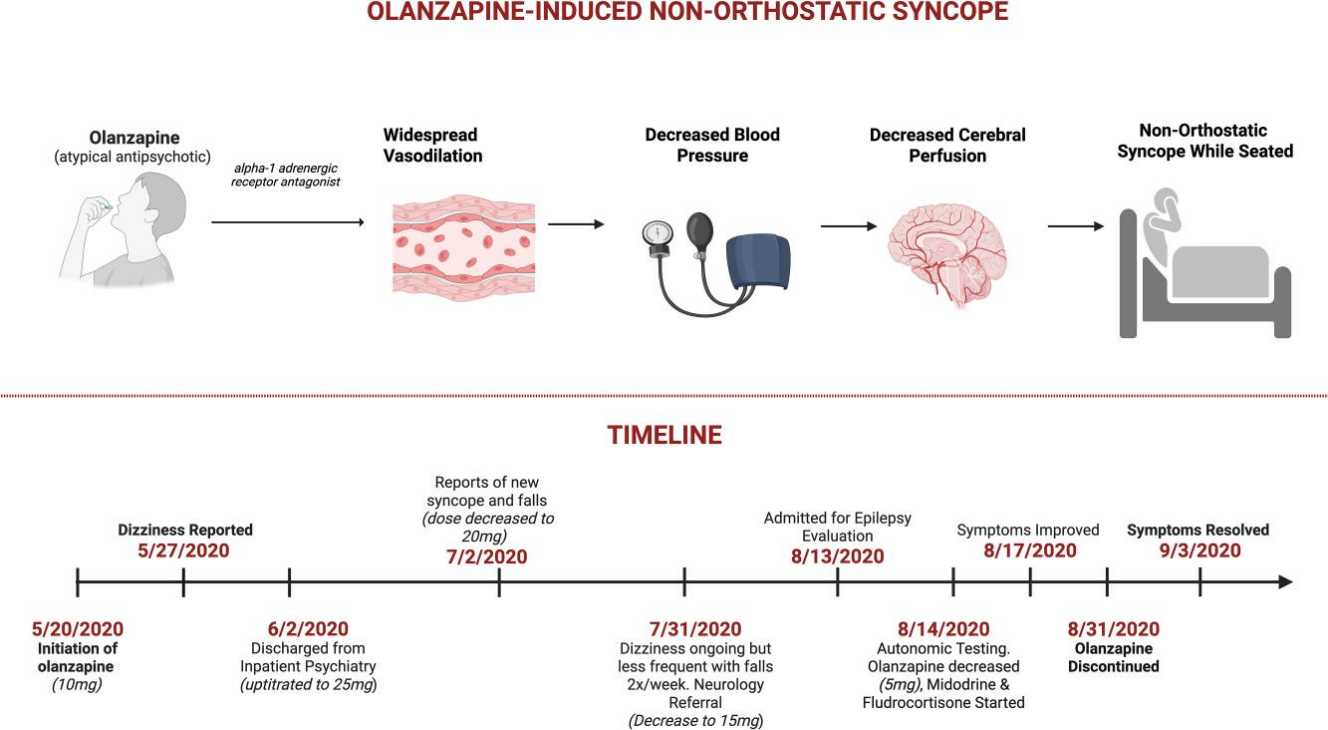


## Case presentation

A 24-year-old male with a history of unspecified psychotic disorder and depression was admitted to hospital for evaluation of recurrent near or complete transient loss of consciousness (TLOC) events. These episodes, occurring up to three times per week, were characterized by light-headedness, tunnel vision, and collapse, with no accompanying autonomic symptoms such as nausea, diaphoresis, or chest pain and no clinical findings to suggest a seizure disorder. The TLOC episodes happened with sudden onset, both while sitting or standing, but often without postural change.

The patient’s medical history included depression, diagnosed at age 14, and a history of alcohol, cannabis, and methamphetamine use, although he was not using any of these agents at time of admission. He reported a history of infrequent and mild transient immediate (initial) OH since his teenage years, which worsened ∼4 months prior to admission after being hospitalized for psychotic and manic symptoms. Notably, during that hospitalization, he was restarted on risperidone (1 mg daily), which he had previously tolerated, and olanzapine (25 mg daily) was newly introduced (*Summary figure*).

Upon admission, physical and neurological exams were unremarkable, and basic laboratory tests, including complete blood count, basic metabolic panel, and urine drug screening, were normal. An echocardiogram showed preserved biventricular function (left ventricular ejection fraction, 60%–65%), and a previous head CT was normal. Common causes of TLOC spells, including vasovagal syncope (VVS), OH, cardiac arrhythmias, seizures, and psychiatric factors, were considered. While VVS is the most common cause of syncope in young adults, the frequency of the patient’s episodes and the lack of typical prodromal symptoms (e.g. nausea and pallor) made this diagnosis unlikely. Extended telemetry and electroencephalogram (EEG) monitoring during hospitalization showed no evidence of arrhythmia or seizure activity, though EEG slowing was noted during the episodes. Based on these findings, the possibility of vasodepressor hypotension was considered, and an autonomic study with concurrent EEG recording was performed.^3^

**Figure 1:**
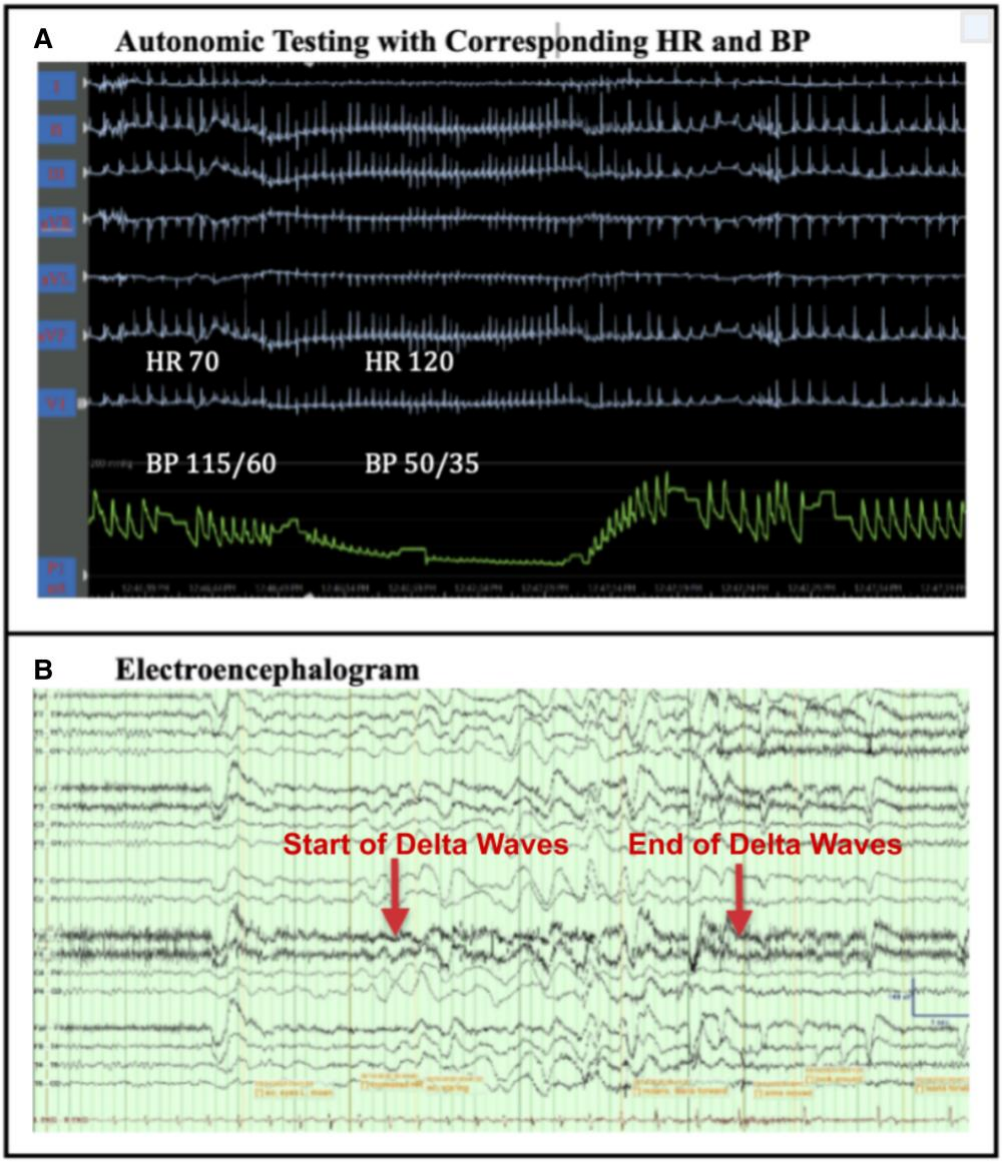
Autonomic testing with corresponding HR, BP, and EEG. (*A*) ECG leads I, II, III avR aVL, aVF, and V1 along with a non-invasive blood pressure trace (P1 art) obtained during a spontaneous hypotensive spell while the patient was seated with legs extended horizontally. He was making no effort to change posture or hold his breath. Marked hypotension occurred followed by spontaneous recovery. Heart rate increased during the hypotensive phase and then slowed moderately when blood pressure normalized. (*B*) Patient sitting up throughout this recording. He moaned softly and leaned forward slightly. About 5 s after the end of this EEG sample, he looked around and pressed the event marker. He interacted normally when staff arrived ∼1 min later. EEG settings: LFF 1 Hz, HFF 70 Hz, notch filter off, sensitivity 7 μV/mm, time base 30 mm/s. EKG sensitivity 50 μV/mm.

During autonomic testing, simultaneous EEG captured multiple TLOC episodes associated with spontaneous-onset paroxysmal hypotension while the patient remained seated with legs extended, without any postural changes (*Figure 1A*). Blood pressures during episodic hypotension were as low as 50/35. During episodes, he bent forward presumably in an attempt to lower his head and improve cerebral perfusion. He did exhibit moderate pallor during TLOC episodes, but no seizure-like activity. These episodes correlated marked EEG slowing (delta waves in *Figure 1B*), a finding previously associated with near-syncope and syncope.^4^ Psychogenic or functional syncope was considered initially; however, the presence of acute EEG abnormalities and concurrent hypotension excluded this diagnosis.^5^

The patient noted that his seated syncopal episodes began around the time olanzapine was introduced. Given the temporal relationship, olanzapine was suspected to be contributing to his symptoms, since it has been reported to trigger OH.^1,2^ The dose of olanzapine was gradually reduced from 25 mg to 5 mg daily, resulting in significant improvement. Four weeks later, olanzapine was discontinued, and risperidone dosing was increased (2 mg). Over the next 3 years, the patient experienced complete resolution of his seated syncopal episodes, although mild OH persisted with prolonged standing (*Summary figure*).

## Discussion

This case provides two new observations. First, our findings suggest that the atypical antipsychotic olanzapine may trigger hypotension in the absence of postural change. While olanzapine treatment has been previously associated with OH, intermittent hypotension unrelated to postural change has not been reported. Second, a primary olanzapine-induced vasodepressor action may in some instances be sufficiently severe to cause symptomatic hypotension without either postural change or evident tachy- or bradyarrhythmia. Beat-to-beat blood pressure recordings such as by non-invasive plethysmographic methods are essential to substantiate a vasodepressor diagnosis in such a situation.^3^

In our case, events often occurred when the patient was seated, and there was no biochemical or haematologic suggestion of volume depletion. Further, the patient received intravenous saline fluid replacement before autonomic testing and despite that intervention, he experienced his typical spells. Finally, the patient underwent detailed neurologic evaluation, and there were no findings to suggest a primary neurogenic cause for his symptoms.

Drug side effects, including OH triggered by antipsychotic medications, tricyclic antidepressants, and antidepressants, are a concern in otherwise healthy patients exposed to these agents.^6^ In our case, a drug-induced mechanism seemed probable as olanzapine is known to cause immediate OH with change of posture.^2^ Furthermore, the patient’s symptoms had started shortly after initiation of this medication and resolved following its discontinuation. While this phenomenon has been observed with olanzapine, it remains unclear whether similar effects occur with other dopamine-serotonin antagonists, as there is currently limited literature addressing this question.

A common mechanism in drug-induced hypotension associated with antipsychotic agents is alpha 1-adrenergic receptor blockade, with direct effect on vascular tone (*Summary figure*).^1,2^ While this adverse effect occurs in only a minority of treated patients (<5%), particularly among older adults, it has the potential to induce dizziness and syncope.^3^ Syncope seems most likely to occur during the initial phase of dose adjustment. Other possible mechanisms include inhibition of centrally mediated pressor reflexes and negative inotropic effects, but these are unlikely in an otherwise healthy young individual.^2^ In addition, other pharmacologic pathways for vasodepressor syncope have been described beyond alpha-adrenergic blockade. These include central nervous system effects seen with beta-blockers (particularly lipophilic agents like propranolol) that can attenuate sympathetic outflow and baroreceptor sensitivity, as well as centrally acting antihypertensives such as clonidine that reduce sympathetic tone through alpha-2 receptor stimulation.^7^ Direct peripheral vasodilation from agents like nitrates, calcium channel blockers, and hydralazine represents another important mechanism.^8^ These diverse pathways highlight the broader spectrum of drug-induced hypotension that clinicians should consider when evaluating syncope, particularly in patients on multiple medications with potential cardiovascular effects.

While there are reports of olanzapine-induced hypotension in the elderly, only one report^1^ has described a case of severe postural OH in a young patient. What makes our case unique is the occurrence of spontaneous hypotension while the patient was seated, without any postural change (*Figure 1*). Further, our case stands apart as we captured multiple episodes of paroxysmal hypotension with EEG slowing even while the patient was in sitting position and we were able to document the hypotension to be primarily vasodepressor in nature (i.e. BP drop without marked HR slowing). Importantly, EEG slowing can result from various causes, including metabolic, structural, or drug-induced aetiologies.^9,10^ However, EEG slowing—especially in the delta range—is often seen with cerebral hypoperfusion when it occurs abruptly during hypotensive episodes.^5,11^ In this case, the rapid onset and resolution of EEG changes tightly linked to hypotension make cerebral hypoperfusion the most likely explanation.

Unanswered at this time, however, is the nature of the trigger that caused onset of the individual spontaneous hypotensive events. One possibility involves circadian variation in autonomic tone—often described as the autonomic ‘clock’—which modulates vascular responsiveness throughout the day. This autonomic regulation typically maintains blood pressure within normal ranges despite varying conditions, but when combined with olanzapine’s alpha-1-adrenergic antagonism, it may create windows of vulnerability where even minor stressors or physiological shifts could trigger profound hypotension without requiring postural changes. The temporal pattern of our patient’s episodes, which occurred unpredictably while seated, suggests such an interaction between autonomic regulation and pharmacologic vasodilation.

Although alpha-1-adrenergic antagonism remains the most plausible mechanism given olanzapine’s pharmacologic profile, alternative explanations such as central autonomic dysregulation or impaired baroreflex sensitivity may also play a role. Central autonomic pathways regulate vascular tone and heart rate variability, and disruption could lead to dysregulated blood pressure control. Similarly, baroreflex dysfunction—if present—can impair compensatory responses to hypotension. However, in this case, formal autonomic testing, including heart rate variability assessment, Valsalva manoeuvre response, and postural blood pressure monitoring, did not reveal significant abnormalities in these regulatory pathways, making these mechanisms less likely contributors, though not entirely excludable. Further research is needed to elucidate the precise mechanisms underlying these episodes and to identify potential risk factors that predispose individuals to such adverse effects. Nonetheless, absence of recurrence after stopping olanzapine points directly to its being an essential causative contributor.

## Conclusions

Olanzapine is known to be a potential cause of OH in the setting of orthostatic stress.^1,2^ However, our observations suggest that olanzapine may also be responsible for a more severe form of seemingly unprovoked intermittent, symptomatic paroxysmal hypotension. Reduction of dose or discontinuation of olanzapine should be contemplated to alleviate symptoms in patients who appear to be experiencing unexplained episodic hypotensive events.

## Lead author biography


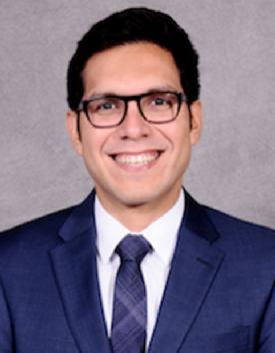


Jorge L. Reyes, MD, MS, is a cardiac electrophysiology fellow at the University of Minnesota. His research focuses on two main areas: the evaluation of syncope and collapse using comprehensive autonomic studies, and the characterization of the relationship between atrial fibrillation and atrial myopathy with cardiovascular and neurocognitive outcomes, such as stroke and dementia.

## Data Availability

To protect the privacy of the individual in this study, the data underlying this article cannot be shared publicly. It will be available upon reasonable request from the corresponding author.
